# Leaving no disease behind: The roadmap to securing universal health security and what this means for the surveillance of infectious diseases in Ghana as a precedent for sub-Saharan Africa

**DOI:** 10.1371/journal.pone.0284931

**Published:** 2023-04-24

**Authors:** Peter N-jonaam Mahama, Amos Tiereyangn Kabo-bah, Giacomo Falchetta, Justine I. Blanford, Edmund Ilimoan Yamba, Prince Antwi-Agyei, Franklin Asiedu-Bekoe, Esi Awuah, Justin Yieri

**Affiliations:** 1 Department of Civil and Environmental Engineering, School of Engineering, University of Energy and Natural Resources, Sunyani, Ghana; 2 Centro Euro-Mediterraneo sui Cambiamenti Climatici, RFF-CMCC European Institute on Economics and the Environment, Università Ca’Foscari Venezia, Rome, Italy; 3 International Institute for Applied Systems Analysis, Schlossplatz, Laxenburg, Austria; 4 Department of Earth Observation Science, Faculty of Geo-Information Science and Earth Observation, University of Twente, Enschede, The Netherlands; 5 Department of Physics, Kwame Nkrumah University of Science and Technology, Kumasi, Ghana; 6 Public Health Division, Ghana Health Service, Accra, Ghana; 7 Department of Civil Engineering, Kwame Nkrumah University of Science and Technology, Kumasi, Ghana; 8 Henson Geodata Technologies, Accra, Ghana; Gunma University: Gunma Daigaku, JAPAN

## Abstract

**Introduction:**

Ghana is the first country in sub-Saharan Africa (SSA) to aim for universal health coverage (UHC). Based on Ghana’s UHC system, the accessibility and distribution of healthcare facilities were evaluated for 2020. Projecting into 2030, this study aimed at providing geographical information data for guiding future policies on siting required healthcare facilities. Ghana as a precedent for SSA was evaluated and proposed to “*leave no disease behind”* in the surveillance of infectious diseases (IDs). This is to reinforce the sustainable development goals (SDG) 3 agenda on health that underpins monitoring equity in “*leaving no one behind*.”

**Methods:**

Geospatial accessibility, travel time data, and algorithms were employed to evaluate the universality and accessibility of healthcare facilities, and their future projections to meet UHC by 2030. Healthcare facilities as surveillance sites were compared to community-based surveillance to identify which would be more applicable as a surveillance system to leave no disease behind in Ghana.

**Findings:**

Ghana has 93.8%, 6.1% and 0.1% as primary, secondary and tertiary healthcare facilities respectively. It has 26.1% of healthcare facilities remaining to meet the SDG 3 health target by 2030. In terms of providing quality healthcare, 29.3% and 67.2% of the additional required healthcare facilities for optimal allocation and achieving the UHC target need to be secondary and tertiary respectively. In assessing the broad spectrum of IDs studied from 2000 to 2020, an average of 226 IDs were endemic or potentially endemic to Ghana. The majority of the studies carried out to identify these IDs were done through community-based surveillance.

**Conclusion:**

Establishing community-based surveillance sites to leave no disease behind and also providing the required healthcare facilities to reinforce leaving no one behind will enhance the universal health security of Ghana as a precedent for SSA.

## 1. Introduction

The risk of a new era of emerging and re-emerging infectious disease (ERID) outbreaks occurring rampantly across the globe is increasing. However, providing healthcare services to all people at risk of an outbreak is often a challenge. This was witnessed by the emergency healthcare structures and interventions that had to be put in place during the peaks of the COVID-19 era in various countries. Providing health services to overcome ID outbreaks depends on the geographical location of the population, the health systems in place, and the spectrum of IDs experienced [1, 2]. In SSA, healthcare resources are already overburdened by the spectrum of IDs faced [3, 4] leaving no room for outbreak situations. Structural adjustment of some SSA countries by the World Bank and the International Monetary Fund (IMF) even made it a mandate to reduce healthcare budgets [5] which limits putting up healthcare facilities. Inadequate healthcare facilities especially in the developing regions of SSA leave it vulnerable to being engulfed by ERID outbreaks. Therefore, the fear is that should there be a virulent pathogen of national or global proportion, some people will be left behind due to their location and inaccessible or unavailable healthcare resources. However, there is a higher chance of saving everyone if every ID is tracked and comprehended in space and time. This can be achieved by leaving no disease behind which reinforces the SDG’s health target of leaving no one behind through UHC.

To track every ID, surveillance capabilities need to be improved. The International Health Regulations (2005) of the World Health Organisation (WHO) recognise disease surveillance as one of the important means to identify, prepare, report and control national and global health threats [6]. To achieve this, many countries collect data from patients seeking care through healthcare facility-based surveillance [7]. However, healthcare facility-based surveillance is challenged by three factors: 1) the marginal proportion of cases that visit such facilities for care [8]; 2) the limited access to care especially in rural areas [9]; and 3) the poor knowledge of healthcare providers about ERIDs [10] which risk unrecognized transmissions. With the knowledge gaps in estimating accurate disease richness from healthcare facility-based surveillance [11], one of the main developments has also been keeping track of publications on IDs [12]. However, as IDs continue to increase in their diversity and spread faster, it is necessary to shift from individually documented patterns of disease surveillance to exploring their host-level surveillance by a well-organised system. Thus, it is necessary to increase surveillance data streams from remote areas where ERIDs are most likely unexplored through community-based surveillance. This will progressively increase access to essential health information for strategic decision-making [13]. In this study, Ghana, the first country in SSA to have implemented the community healthcare system aimed at UHC was used to evaluate the universality and accessibility of healthcare facilities. The use of these healthcare facilities as surveillance sites was also evaluated. Their ability to reinforce leaving no one behind by proposing the surveillance of the broad spectrum of IDs as means to leave no disease behind was also evaluated. This proposed integrated approach would allow eliminating the systemic barriers that leave people behind across geographic units and meeting the SDG’s health target if effectively implemented [14].

## 2. Datasets and methodology

### 2.1. Datasets

The study was carried out using two main datasets, the healthcare facilities across Ghana from the Policy Planning Monitoring and Evaluation Division (PPMED) of Ghana Health Service (GHS), and the database of peer-reviewed articles on IDs of Ghana from the Global Infectious Diseases and Epidemiology Network (GIDEON). A breakdown of the datasets used for the study is shown in Table 1.

**Table 1:** Datasets used for the study and their sources.

Dataset	Unit	Spatial Resolution	Source
Infectious diseases of SSA	Count	Country-level	[3]
Infectious diseases of Ghana	Count	Regional-level	[3]
Healthcare facilities in Ghana	Typology	Exact position	PPMED—GHS
Ghana’s population shapefile and its administrative units	-	Regional to district level	Ghana Statistical Service
Global Fraction Surface for travel speed	Minutes/meter	1 km	[15]
Global human settlement model grid (GHS-SMOD) 2015 for settlement classification	Class	1 km	[16]
Population and urbanization projections to 2030	Count and share	Regional-level	[17]

The 9,011 health facilities as of the year 2020 from 1,394 sub-districts across the country were obtained from PPMED-GHS on request. The database has each healthcare facility with 8 descriptive details–region, district, sub-district, facility name, ownership, facility type latitude and longitude. The ownership constitutes facilities that belong to the government, quasi-government, mines, Christian health association of Ghana (CHAG) and other faith-based, and private individuals and organisations. The coordinates are given in decimal degrees format in the World Geodetic System 1984 (WGS84) coordinate system. In terms of the type of facility, they were grouped into three based on the similarity in functions and the different levels of service delivery since Ghana runs a referral system which is interlinked from primary (8,452 facilities) to secondary (552 facilities) and tertiary (7 facilities). Since it was the same country, a primary healthcare facility will have similarities in functions across the country as the other types. Primary-level healthcare facilities serve as the first contact of the target population within communities and sub-districts. These constitute the Community-based Health Planning and Services (CHPS) compound, health centres, clinics, and polyclinics. The secondary level of healthcare facilities serves as the second referral point from the primary care and are facilities that are located at the district and regional levels. These include hospitals, district hospitals, regional hospitals and university hospitals. The third category of the healthcare system is a well-established teaching hospital and specialised hospitals.

On the other hand, the country series data of the GIDEON database was used to obtain the number of endemic or potentially endemic IDs of the SSA countries [3]. The GIDEON databases are based on standard textbooks, peer-review journals backed by scientific evidence, Health Ministry reports and ProMED, supplemented by an ongoing search of the medical literature through an online platform [18]. Information from these materials is extracted to form the GIDEON database for each country. For each of the SSA countries, the total number of endemic or potentially endemic IDs was obtained for further analysis. For Ghana, the broad spectrum of its IDs was extracted. A detailed description of the extraction of data from each article, and the inclusion and exclusion criteria are presented in Mahama et al. [19]. This data consists of extracted data from articles covering the type of survey for the sampling or study, the infectious disease, month and year of sampling or study, region, district, town, facility, study group, percentage studied or results, month and year the article was published, the journal, and title of the article. The interest within this dataset was the healthcare facilities. This indicated which healthcare facility each surveillance study was carried out for that research. The remaining surveillance studies were within community settings.

Details of how Ghana’s population shapefile and its administrative units were obtained and georeferenced are captured in Mahama et al. [19]. Details of the Global Fraction Surface for land-based travel speed, global human settlement model grid (GHS-SMOD) 2015 for settlement classification, and population and urbanization projections to 2030 are described in Falchetta et al. [20].

### 2.2. Analysis

#### 2.2.1. Accessibility and universality of healthcare facilities in Ghana

**Fig 1:**
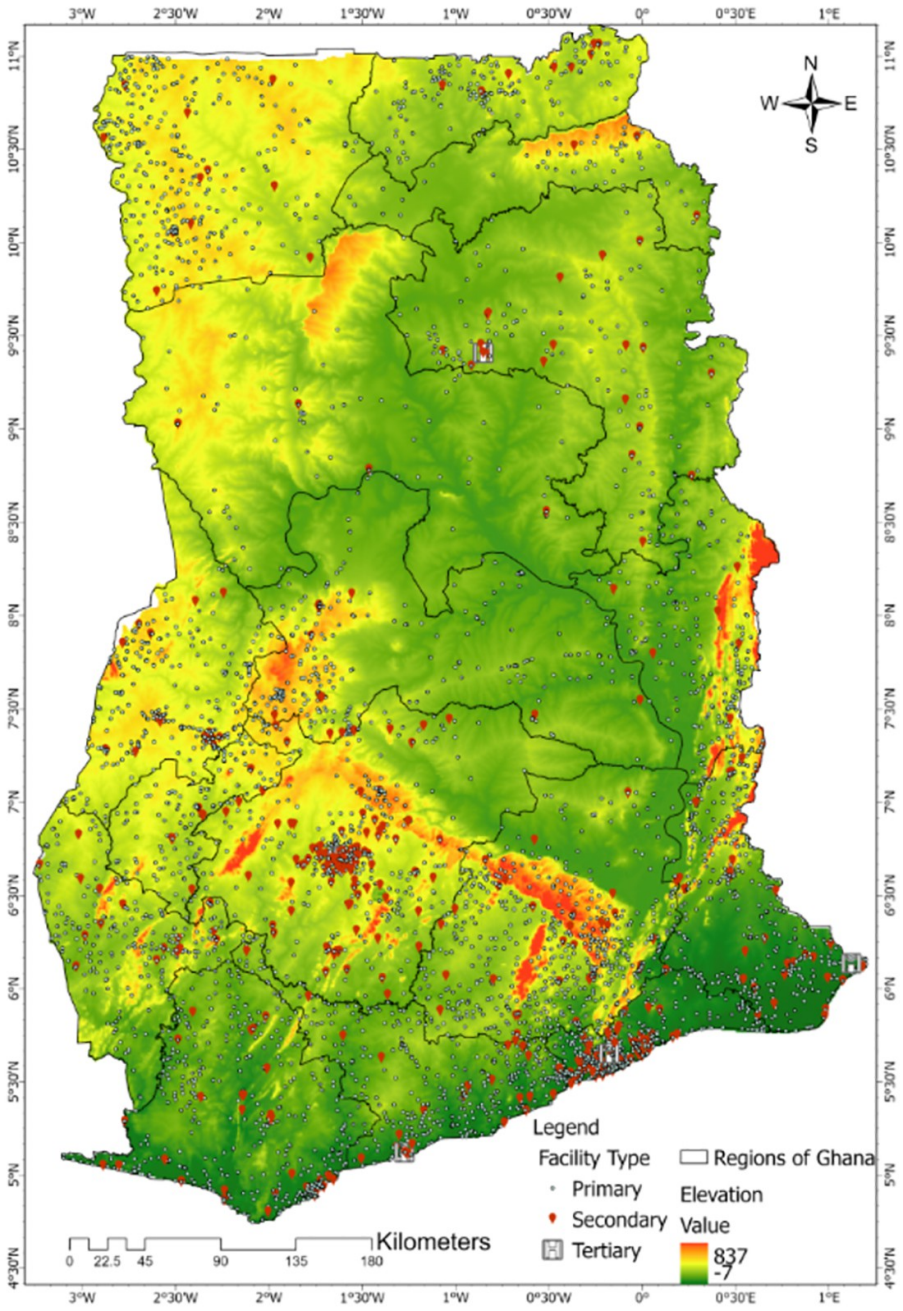
Distribution of primary, secondary and tertiary healthcare facilities across Ghana as of the year 2020. This also shows the digital elevation model of Ghana.

Out of the 9,011 healthcare facilities, the coordinates in decimals of 7,299 (81.0%) were obtained. The distribution of healthcare facilities and the digital elevation model of Ghana are shown in Fig 1. Some of the healthcare facilities were randomly selected using the facility’s name and/or sub-district name and verified by visual inspection in Google Earth. Facilities that had their coordinates plotted in wrong districts, water bodies and outside the country boundaries were assigned new coordinates by doing a minor shift in QGIS. Facilities without coordinates (19.0%) were then excluded from the accessibility analysis. Timely access to healthcare for a target population is essential to provide the desired health outcomes. Using two measures of universality and accessibility, the distance and travel time to healthcare facilities by a target population were analysed.

First, based on the 5 km radius of a healthcare facility to the target population recommended by the World Health Organisation (WHO), the catchment zone of the healthcare facilities was analysed using QGIS to determine the spatial distribution and/or universality. The facilities’ coordinate points, projected shapefile of the country and its regions, and road network (although not used) were added. The geometry of the entire area (EA) and the entire population (EP) were calculated and placed in new fields. Using the buffer geoprocessing tool, the points with the 5 km radius buffer were created. The buffer and the population were then intersected to the shapefile. The newly created shapefile was then projected. The geometry of the intersected area (IA) was then calculated. A new field was then created to calculate the proportion of the intersected population (IP) that falls within the buffer using ‘Field Calculator’ as shown in Eq 1.

$$IP=\frac{IA}{IE}\;\times EP$$ (1)

Second, based on the travel time the proportion of the population living within an estimate of 30, 60 and 120 minutes to the nearest healthcare facility was analysed using RStudio. Two main datasets; a 1-km-resolution fraction surface layer of Ghana and the healthcare facilities points data were used. The fraction surface layer contains for each pixel the overall speed of travel in minutes per meter. The fastest mode of travel is retained given the infrastructure and barriers for each pixel and in moving from one cell to the adjacent ones. Using these two datasets and the shapefile of Ghana, the travel time accessibility is estimated using the procedure of Weiss et al. [15] as presented in the code and data described in Falchetta et al. [20]. With the increased amount of data from an improved national health information system, the accessibility modelling and maps were upgraded from the global [15] to sub-Saharan Africa [20] to the country level (Ghana) as presented in this study. This followed the procedure by converting the 1-km-resolution fraction surface layer into a transition matrix, georeferencing the healthcare facilities location and using the accumulated cost algorithm from the gdistance package in R to generate a raster layer of the travel time (in minutes) to the nearest healthcare facility. Thus, estimates of the accessibility were based on the population living within the given travel time to the nearest healthcare facility and visualised using an empirical cumulative distribution function to determine the fraction of the population within a given travel time for each type of healthcare facility [16]. To identify the spatial land-use plan [17] and for population and urbanization projections, the healthcare facilities required to meet the universal accessibility needs of Ghana by 2030 were then estimated and presented as a map. This was first by assessing whether the country had been able to meet its target of attaining the UHC and bridging the accessibility inequity gap as of 2020. Else, a projection of healthcare facilities that will be required to meet SDG 3 by the year 2030 was required. This was by identifying the optimal spatial allocation of the minimum number of new healthcare facilities that will allow the entire population of Ghana to access healthcare within a range of a maximum of 30, 60, and 120 minutes from the nearest healthcare facility in primary, secondary, and tertiary respectively as applied in Falchetta et al. [20]. A detail of the GIS routine and optimizations applied can be found in Falchetta et al. [20].

#### 2.2.2. Leaving no disease behind through surveillance

As of the year 2020, the intensity and distribution of endemic or potentially endemic IDs of SSA were assessed using the GIDEON database. The 49 countries used were categorised into intensity classes (IC) from 190 to 250 with an interval of 20 IDs. These intensity classes were based on the range of their endemicity: IC1, 190–210; IC2, 211–230; and IC3, 231–250. The spatial distribution of the IDs was also grouped into distribution classes (DC), based on the sub-regions: DC1, West Africa; DC2, East Africa; DC3, Central Africa; and DC4, Southern Africa.

Scaling down, the GIDEON database was then used to analyse the number of IDs studied and present in Ghana from 2000 to 2020. The IDs studied in 196 out of 260 districts of Ghana were extracted from peer-reviewed articles over the period. These studies at the district levels were then aggregated into the respective 16 regions of Ghana. The type of surveillance system used in each study was then identified and grouped into healthcare facility-based and community-based surveillance. These surveillance types were then evaluated using a pie chart represented in each region. Healthcare facility-based surveillance was based on studies done through human samples taken from individuals within healthcare facilities. Community-based surveillance constitutes humans, animals and their products, water, food, vegetables, insects, birds and soil samples. Research samples of each category were aggregated from the district to the regional level. The prominent form of surveillance over the period was then proposed as the system to survey all the IDs in Ghana in leaving no disease behind. Herein, further discussions on the geospatial health security and state capacity, and response capabilities to leaving no disease behind in Ghana as the proposed pilot study for SSA.

## 3. Results

### 3.1. Accessibility and universality of healthcare facilities in Ghana

**Fig 2:**
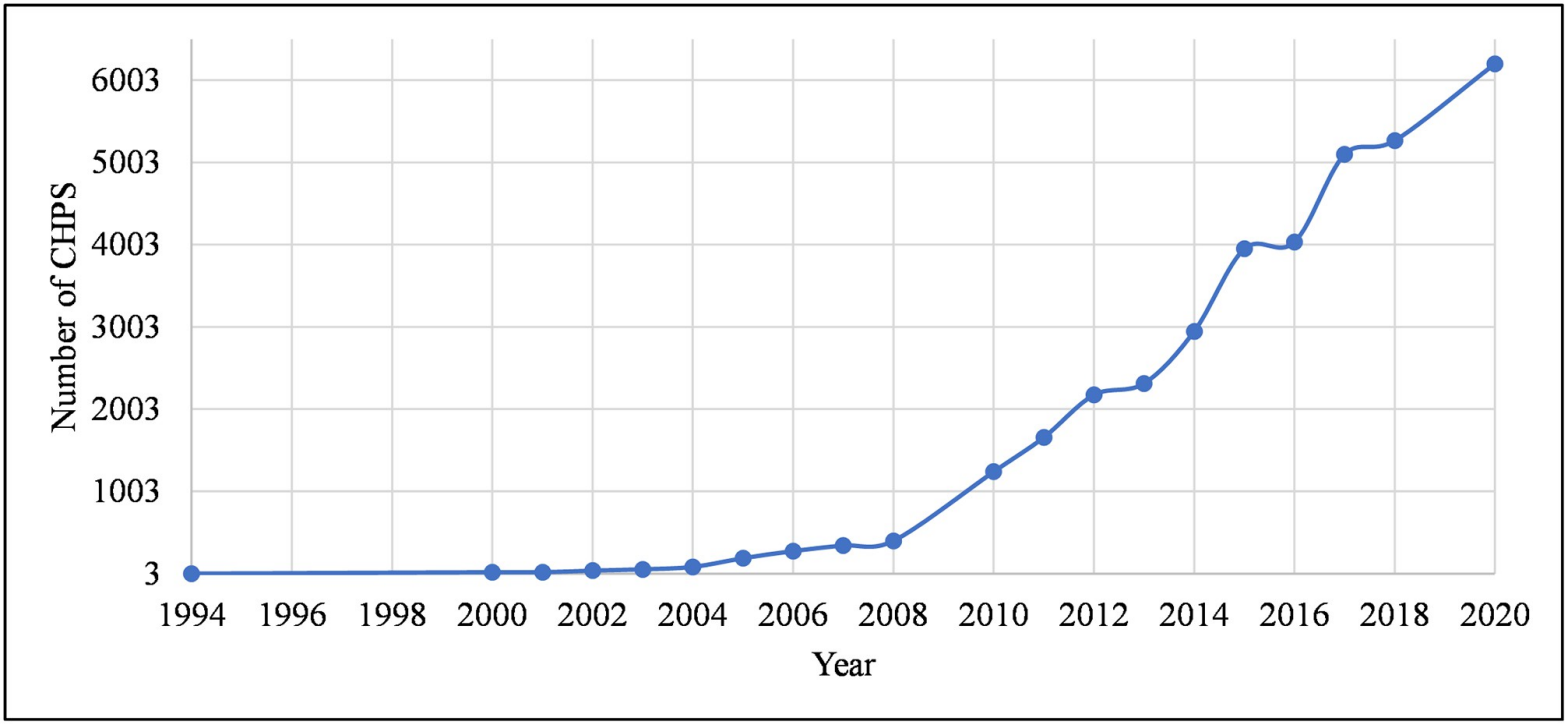
The number of functional CHPS over the years. The results are from its three-village experimental phase in February 1994 as the first African demographic impact of aligning community health services with traditional social life and institutions [23] through documents [24, 25] to the last updated version of the dataset used for this research in the year 2020 by the PPMED-GHS.

Achieving UHC has been a global health campaign. It is widely accepted in many places and appears ready for scale-ups for its new SDG health target by the year 2030. However, visible to its implementation challenges is that a large part of the population in low- and middle-income countries are yet to pass the proof-of-concept phase. In Ghana, the introduction of the CHPS as a target to deliver care directly to communities to bridge the equity gaps in healthcare across the country has served as the main leverage for UHC. This was adopted from Bangladesh in 1994 with the Navrongo Community Health and Family Planning Project as the model experimented to bring essential health services close to the community [21]. Before that, the concept of “*Health for All*” by the year 2000 was envisioned at the Alma Ata conference in 1977 to be implemented by the Ministry of Health as a global mandate [1, 22]. The pilot implementation of CHPS in three sub-districts through to the year 2020 is shown in Fig 2.

After the implementation of CHPS, the delivery of health services at the district level was thus pegged to have a three-tier level starting with CHPS for the community level, health centre for sub-district and hospital for the district level. This healthcare system designated at the district level also respectively served as the referral system for health delivery services at that level. The demarcation of CHPS has been based on electoral areas or communal zones which constitute a geographical area of a 4-kilometre radius and between 4,500–5,000 persons or 750 households mostly of rural settings [21]. By the year 2020, Ghana could boast of 6,181 CHPS and 2,830 other healthcare facilities with its regional breakdown in Table 2. Although the service provided may have similarities based on the level of care, the quantity and type of healthcare infrastructure vary across regions. The private sector (CHAG and other faith-based, mines, private and quasi-government) healthcare facilities form a significant number of the facilities (17.0%) compared to the government-owned facilities (83.0%) across the country.

**Table 2:** Regional breakdown of the types of healthcare facilities in Ghana as of the year 2020.

Region	Primary Healthcare Facilities (CHPS)	Primary Healthcare Facilities (Others)	Secondary Healthcare Facilities	Tertiary Healthcare Facilities
Ahafo	129	43	10	-
Ashanti	1111	350	164	1
Bono	303	121	18	-
Bono East	281	66	13	-
Central	445	173	29	1
Eastern	841	223	41	-
Greater Accra	719	451	127	3
North East	96	28	4	-
Northern	310	107	33	1
Oti	173	47	7	-
Savannah	118	43	4	-
Upper East	362	113	12	-
Upper West	322	94	13	-
Volta	317	161	28	1
Western	404	187	33	-
Western North	250	64	16	-

**Fig 3:**
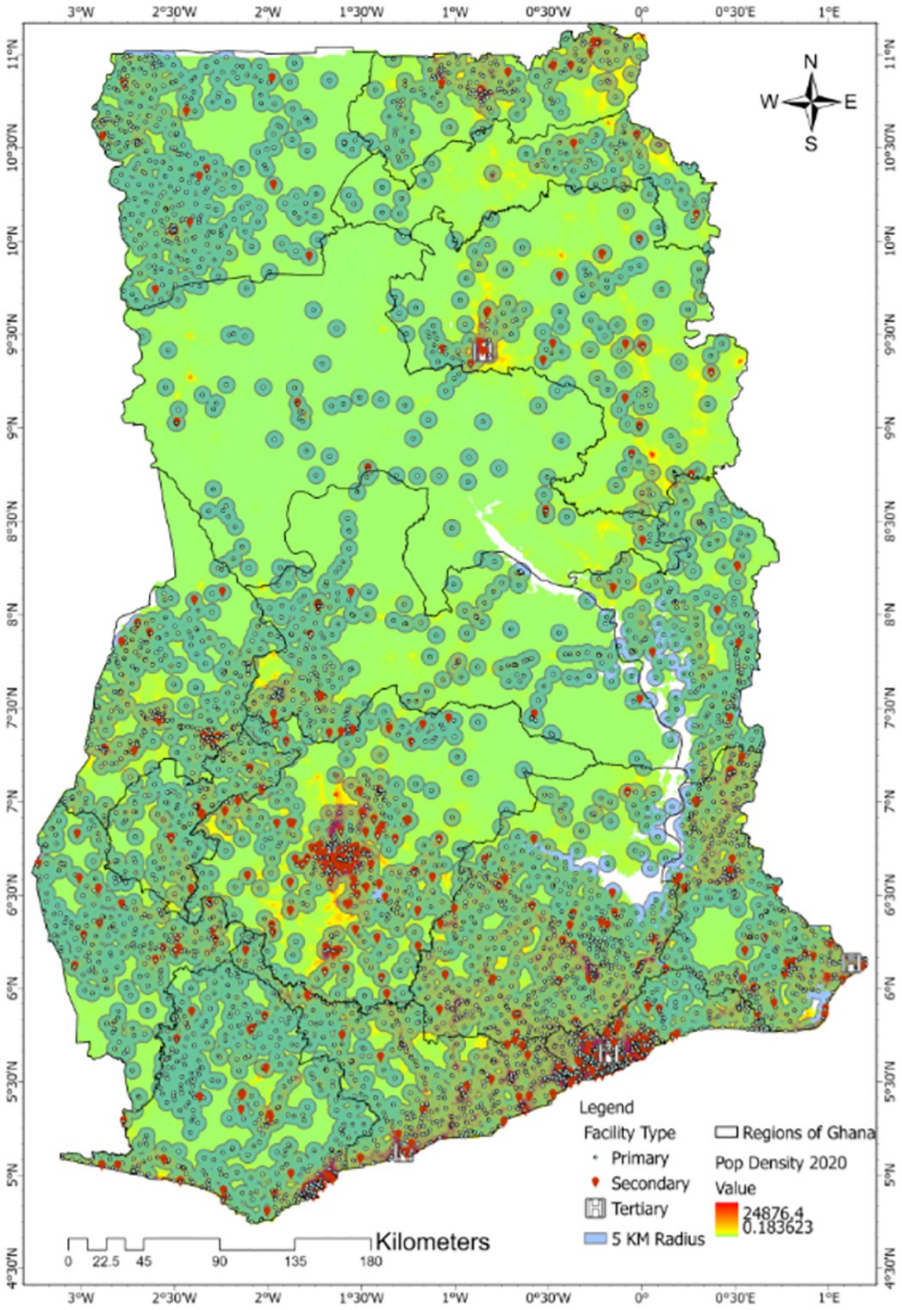
The accessibility catchment zone of healthcare facilities in Ghana based on the 5 km radius recommended for optimal access and population-to-facility ratio by WHO. The 5 km buffer of each healthcare facility was developed on the population density at the year 2020 to ease visual analysis of the catchment areas.

**Fig 4:**
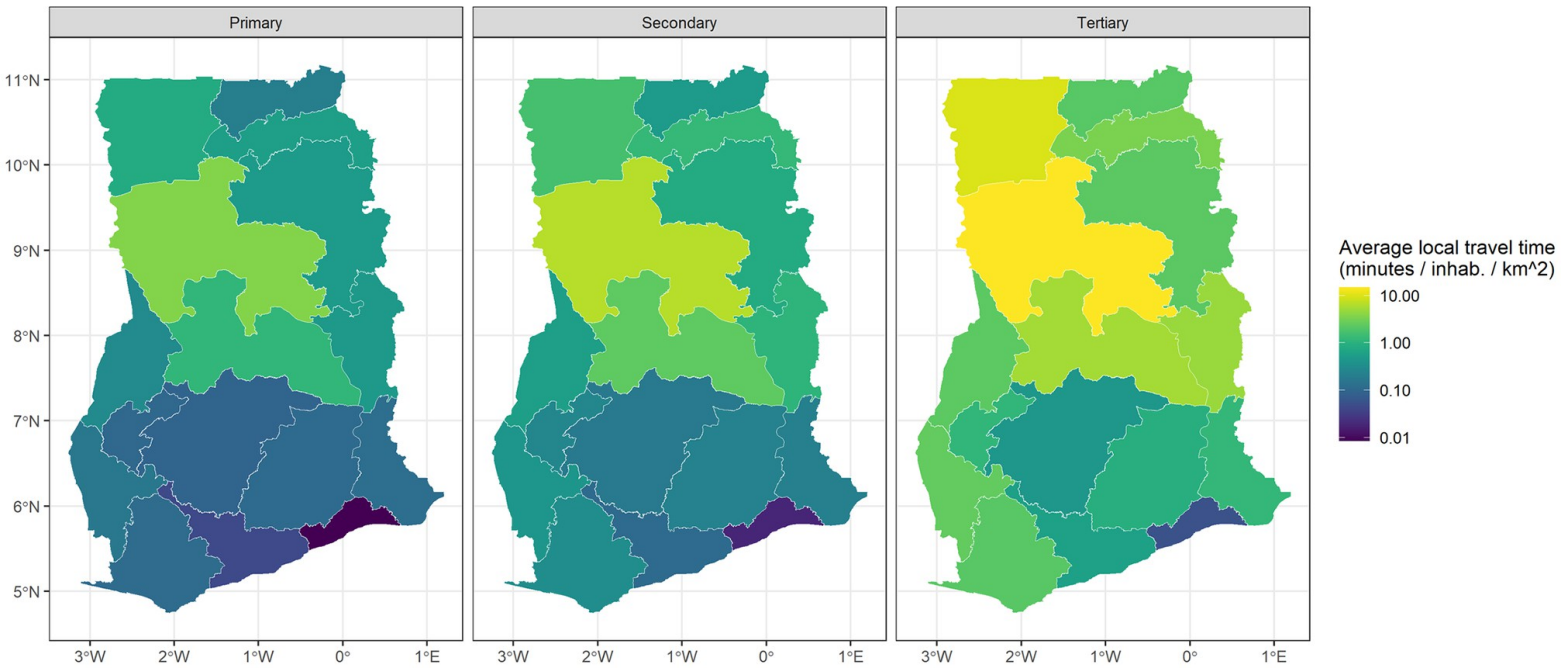
The accessibility of healthcare facilities type (primary, secondary and tertiary) in travel time of the 16 regions of Ghana. The results are logarithmically transformed.

**Fig 5:**
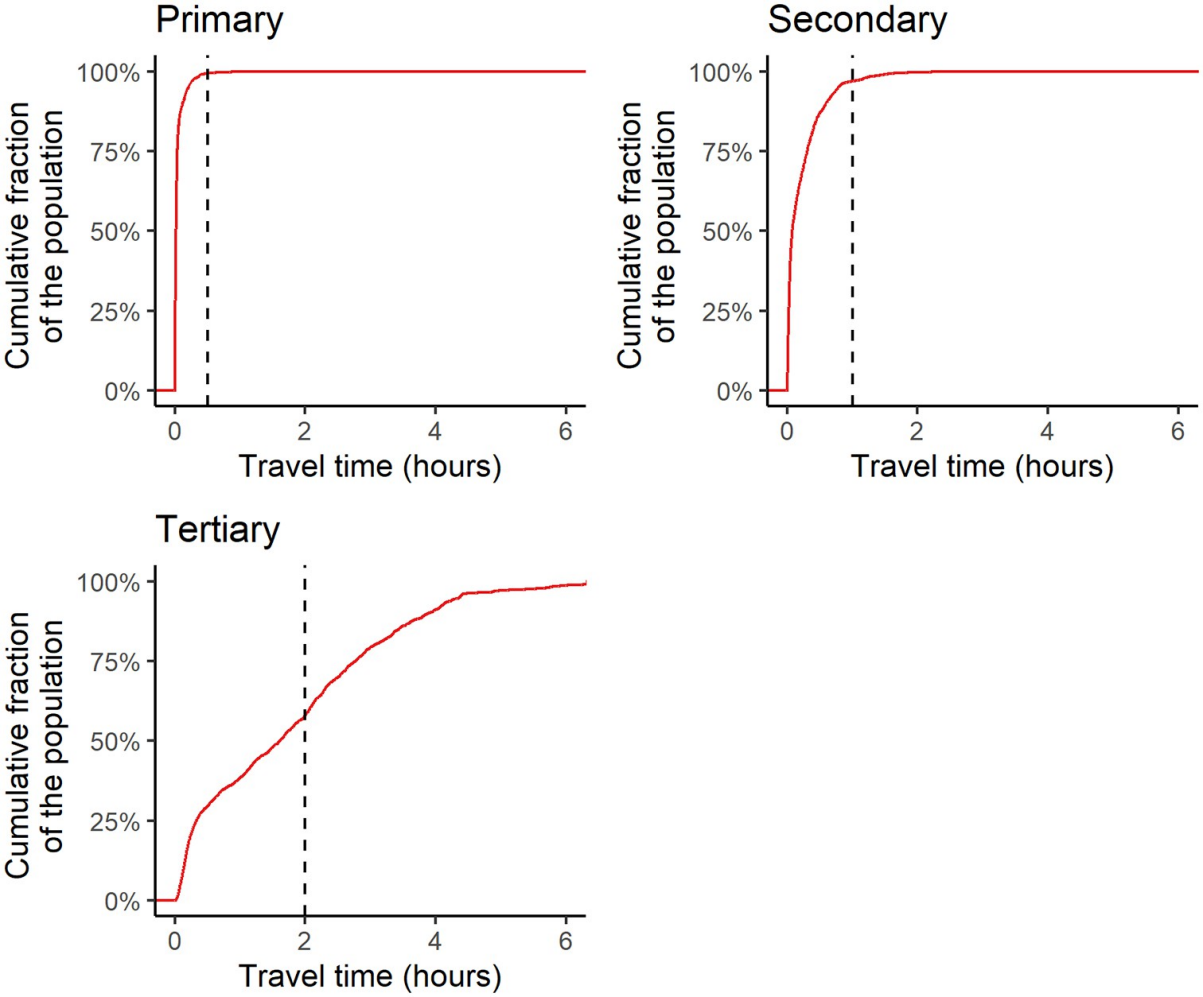
The empirical cumulative distribution curves of the population’s accessibility to the nearest healthcare facility type. The x-axis describes the travel time to the healthcare facility type in hours. The y-axis describes the cumulative fraction of the population that is living within the travel time of the corresponding x-value.

Except for maternity homes and psychiatric hospitals that provide specific healthcare which is not clinically disease-based treatment centres, the rest of the healthcare facilities were categorised into primary, secondary and tertiary. This was compared to the standard of categorisation in the sub-Saharan Africa region as presented in Maina et al. [26]. Based on the population-weighted 5 km radius buffer, a map of healthcare facilities’ catchment areas was developed as shown in Fig 3. Using the average travel time to the nearest healthcare facility in each region, accessibility maps as shown in Fig 4 were developed. These maps indicate that the travel time increases geographically from the south to the north, and demographically from primary, secondary to tertiary healthcare facilities. The healthcare facilities across the country as categorised were 93.8% primary, 6.1% secondary and 0.1% tertiary. Using the 5 km radius recommended optimal access and population-to-facility ratio by WHO as shown in Fig 3, the population to the healthcare facility type were 25,077,239 for primary, 3,071,337 for secondary and 37,215 for tertiary in the year 2020. Using the travel time of 30, 60 and 120 minutes respectively, the empirical cumulative distribution curves of the population’s accessibility to the nearest healthcare facility type were 99.4% (primary), 96.9% (secondary) and 58.1% (tertiary) at the year 2020 as shown in Fig 5.

By visual analysis, it was observed from the results that the healthcare facilities were concentrated in areas with high population density as seen in the Greater Accra, Ashanti and Eastern regions. For primary facilities, these three regions have 43.7% whereas the remaining thirteen regions have 56.3%. For secondary healthcare facilities, these three regions have 61.2% whereas the remaining thirteen regions have 38.8%. Out of the 7 tertiary healthcare facilities in the country, the Greater Accra Region alone has 3. The least regions with accessibility to all three types of healthcare facilities were geographically within the northern part of the country. All these analyses were carried out using a modest approach to define healthcare facility catchment areas based on principles of least effort and distance decay where people turn to access healthcare services based on proximity to general services. However, people may bypass the nearest facilities to access healthcare elsewhere based on the complexity and/or speciality of care required [27].

**Fig 6:**
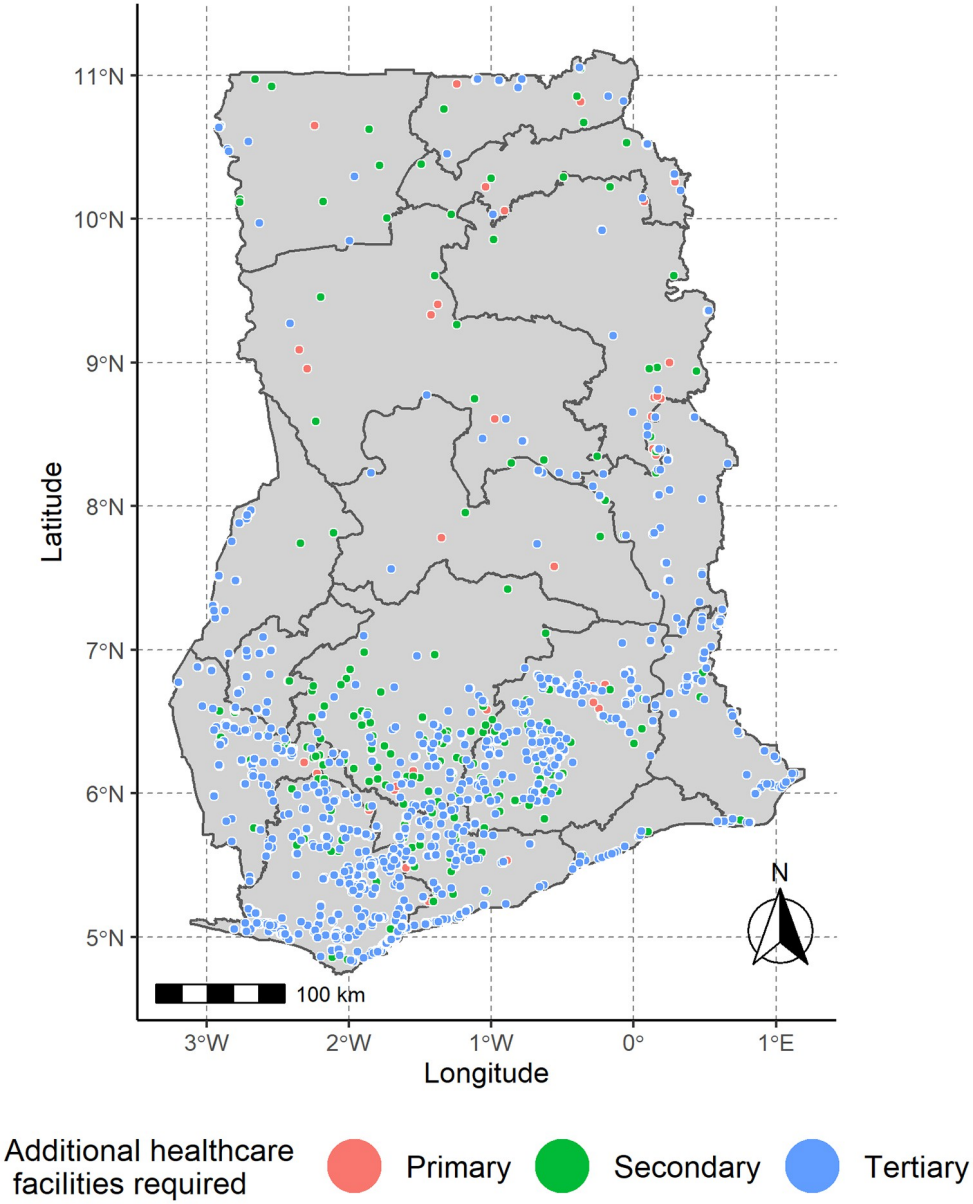
Map of healthcare facilities required to meet the SDG 3 target in Ghana by the year 2030. This shows the predefined travel time of 30, 60 and 120 minutes for primary, secondary and tertiary which are respectively colour coded in ensuring the optimal location and accessibility of healthcare facilities in Ghana.

Further analysing the universality of healthcare facilities in Ghana, after assessing the accessibility inequity gap as of year 2020, a projection to meet SDG 3 by the year 2030 is indicated as shown in Fig 6. This indicates that the number of additional healthcare facilities needed to meet the required travel time of 30, 60 and 120 minutes respectively for primary, secondary and tertiary are 90, 757 and 1,733. This means, more quality healthcare facilities (29.3% and 67.3%) for secondary and tertiary compared to 3.5% for primary are needed. In terms of the universality of healthcare facilities, Ghana has covered 73.9% remaining 26.1% to make up for the SDG 3 health target of leaving no one behind in terms of accessibility.

### 3.2. The distribution and intensity of infectious diseases in sub-Saharan Africa

Out of the globally 361 generic IDs recognised by the GIDEON database, a breakdown of the endemic or potentially endemic IDs are as shown in Table 3 for SSA by 2020. This indicates that the SSA region which occupies less than 20% of the earth’s total land area and is home to 14% of the world’s population has as high as 69% of endemic or potentially endemic IDs recognised in the world today. From the intensity classes, 8 countries (16%) were within IC1, 27 countries (55%) in IC2 and 14 countries (29%) in IC3.

**Table 3:** Regional breakdown of endemic or potentially endemic infectious diseases of Sub-Saharan Africa (SSA). It is categorised into distribution classes (DC–West Africa (1), East Africa (2), Central Africa (3) and Southern Africa (4)), and intensity classes (IC– 190–210 (1), 211–230 (2) and 231–250 (3)). The results were obtained from data worked from the GIDEON 2020 country series database.

Parameter	DC1	DC2	DC3	DC4	IC1	IC2	IC3
Number of Countries	16	16	8	9	8	27	14
Minimum	202	193	208	202	193	211	231
Average	224	222	227	216	202	221	237
Maximum	249	245	237	231	208	230	249

**Fig 7:**
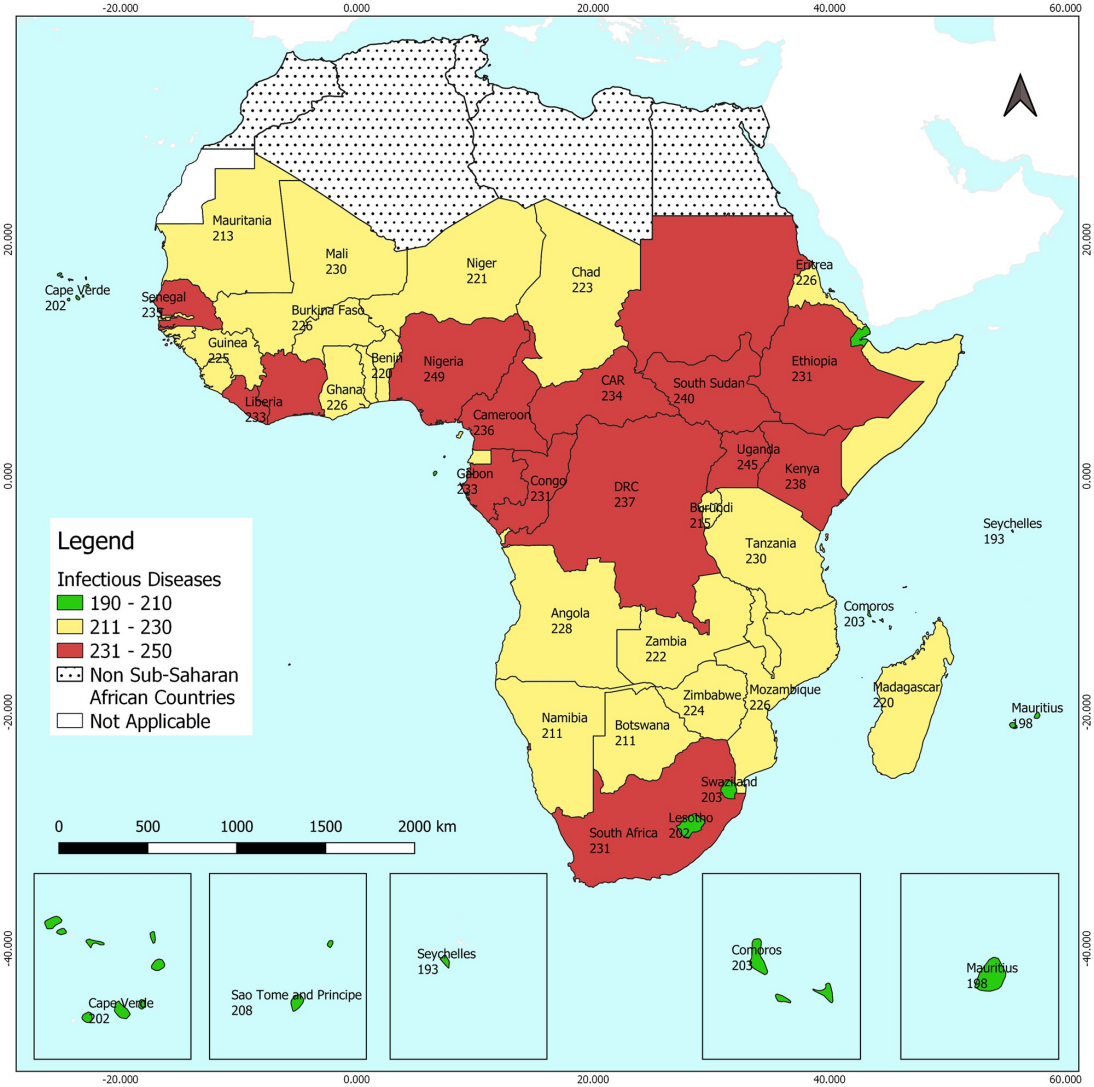
This map represents the endemic or potentially endemic infectious diseases of SSA in 2020. The intensity map which is colour coded accordingly ranges from 190 to 250 with intervals of 20 infectious diseases.

The highest minimum number of IDs of the sub-regions as depicted in Table 3 also indicated that these diseases are more concentrated in the Central Africa region as shown in Fig 7. The lowest number of endemic or potentially endemic IDs in SSA based on the intensity distribution is in Seychelles and Mauritius (island countries of East Africa) and the highest is in Nigeria and Uganda within West Africa and Central Africa respectively.

### 3.3. Surveillance of infectious diseases in Ghana

**Fig 8:**
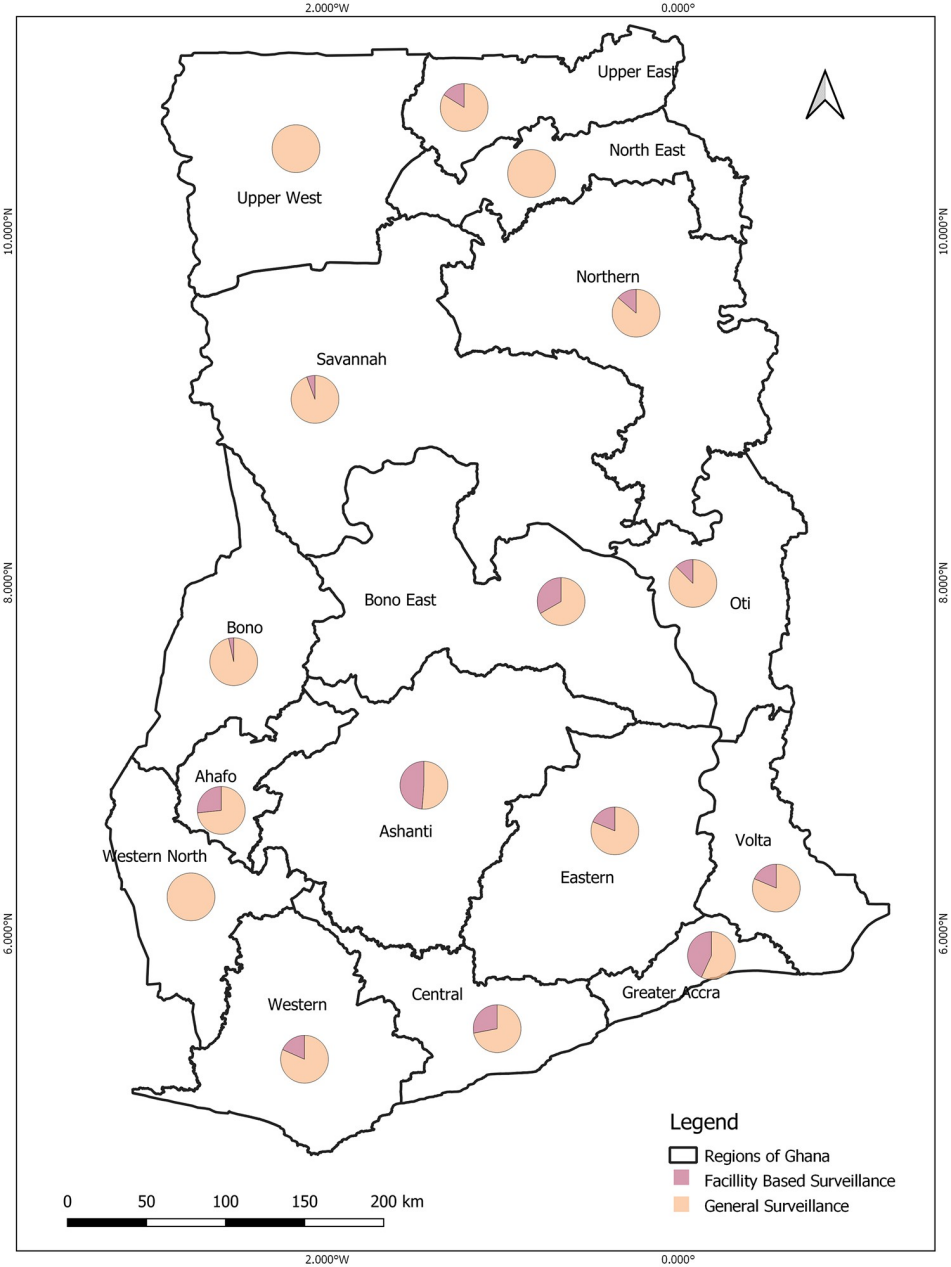
Comparing healthcare facility-based surveillance versus community-based surveillance in the various regions of Ghana using pie chat. This is based on published peer-reviewed articles on IDs from the year 2000 to 2020 extracted from the GIDEON database.

The estimates for healthcare coverage go beyond numbers that aim to “*leave no one behind*.” Several questions still pop up to improve health coverage. One such which needs extensive studies is which group of people are being left behind? Although Ghana might boost a good coverage of CHPS which constitutes about 66% of healthcare facilities across the country, does it have the essential healthcare coverage needed to stand the trend in ERIDs? As a country within a sub-region prone to ERIDs, there is the need for an effective surveillance system as the basis for not just answering these questions but to know which diseases are circulating in the country. If for nothing at all, the COVID-19 pandemic has thought that some people will be left behind if things grow out of global or national capacity. However, the clinical setting of CHPS which forms the majority of healthcare facilities makes it difficult to detect ERIDs. Most of the other facilities are not also upgraded to run timely and reliable detection of these ERIDs. Healthcare facility-based surveillance is therefore critically challenged and unsuitable as a standalone primary source of detecting ERIDs in resource-limited settings such as Ghana. From the results, as shown in Fig 8, community-based surveillance on IDs was conducted more compared to healthcare facility-based surveillance over the 20 years studied. Regions such as the Upper West, North East and Western North did not have facility-based surveillance.

## 4. Discussion

### 4.1. Universal health coverage in Ghana

From the results, the introduction of CHPS has significantly improved healthcare accessibility with the number of such facilities established across the country. However, some limitations in the equity in geographical access to quality healthcare services is a challenge. Thus, resources and effort would have to be invested to retool humans and infrastructure across the country if such primary facilities are to have diagnostic and surveillance capabilities. First cases of ERIDs can easily elude such healthcare facilities since they are not well resourced to detect them. The demand for well-established healthcare facilities of secondary and tertiary standards is thus required to be located in strategic geographical areas across the country as presented in the results. Despite the data limitations, the estimate of the number of healthcare facilities needed to meet the healthcare requirement by 2030 indicated that there are a lot of people still left behind. The number of secondary and tertiary healthcare facilities that can provide basic and special services are concentrated in cities and are few. Thus, to provide special care to patients, especially with the outbreak of an ERID the country would not have the capacity to provide effective and universal care as envisioned. It would turn out to be that the urban areas are well resourced leaving the rural areas to struggle with their health needs. To achieve equity would mean upgrading most of the primary facilities to secondary facilities and the secondary facilities into tertiary or setting up new facilities as presented in the results. Since this is just an upgrade of physical accessibility, it will require having to provide trained professionals, equipment, communication and transportation to meet a certain preparedness level to be able to withstand unforeseen circumstances from ERIDs. Achieving this in a developing country such as Ghana might take years beyond the targeted 2030. The rural areas especially are not privileged to secondary healthcare facilities for emergencies and even services that require timely and appropriate care compared to urban areas. This disproportionality affirms the inverse care law where those who need much care are not given and those who need less care receive it in abundance. Therefore, it is fair to all to demand leaving no disease behind so the disadvantaged would not be left alone to battle with them.

From the results, variation in the travel time to the nearest healthcare facility can be related to the heterogeneous land cover and population density across the country. The northern part of the country has vast land cover compared to the southern sector. This inversely correlates with the population density, urbanisation and socio-economic development. Thus, the healthcare facilities of the northern sector were less dense as presented in the results. Although these areas are less endowed and/or dense with healthcare facilities, it would be unfair to conclude that rural areas have farther healthcare facilities since some cities also have unequal distributions of healthcare facilities. The resulting projection into 2030 showed the estimated healthcare facilities required for the northern sector still less dense for major sections compared to the southern part of Ghana. The positive trend between large travel time and underserved populations indicates the disparity in people that would be left behind in healthcare accessibility.

In terms of limitations, 19.0% of the healthcare facilities did not have their coordinates and were excluded from the analysis and therefore constituted a data-related limitation that can be included in future analysis. The density of the healthcare facilities in urban centres especially Accra resulted in overestimating the accessibility in such areas. This caused duplication of the population of people within the catchment area since they appear in several buffers covering the same area. Also, because of the densification of urban areas resulting in changes in spatial planning and the possible expansion of primary facilities into secondary facilities, the travel time that applies to these results might not reflect in the year 2030. Whereas the development of some structures such as roads and more healthcare facilities might increase accessibility, other factors such as poor road conditions, overwhelmed transportation system and urban development might rather affect their efficiency.

### 4.2. Why advocate to leave no disease behind in Ghana as a precedent for sub-Saharan Africa?

Generally, the burden of IDs varies by country, partly because of differences in vaccine distribution and access to care, geography, climate, crowding, nutritional status, travel, and possibly genetic differences in populations that affect disease severity. The results revealed that the share size of endemic or potentially endemic IDs that plague the SSA region is of a magnitude that cannot be overlooked as it possesses health security threats of different dimensions. It would not be overrated to peg each person in SSA as haven had at least one ID in a lifetime. From the results, SSA which is still the dominant hub of global deaths and Disability-Adjusted Life Years [28] is attributed to being plagued with as high as 249 and average of 222 IDs by the year 2020. Judging by the trend, the period of the futuristic SDG health target of 2030 and Africa’s 2063 agenda could be plagued with over 300 endemic or potentially endemic IDs if the calls to invest and fortify preparedness, control, and surveillance are not herded. The share size of IDs reported from the SSA region could be due to its rapidly changing social and demographic characteristics that favour their proliferation. The extent of climate change coupled with poor water, sanitation, environmental and living conditions; weak health systems; inadequate surveillance capabilities and endangered health research leaves it vulnerable to the spread and complete denial of response to these diseases. Ghana been the pacesetter to UHC in SSA, it is advocated that it serves as a precedent to implement the principle to leave no disease behind.

## 5. Conclusion

Ghana is the first African country to align community health services with traditional social life and institutions aimed at achieving UHC. To reinforce domestic concerns on the SDG 3 health target, the study evaluated the accessibility and distribution of healthcare facilities based on Ghana’s UHC system. The study revealed how important to have universal access to healthcare and equitable distribution of such services. It also showed the estimates of people who lack access and the number of healthcare facilities required across the country. To guide future policies on siting healthcare facilities, the study evaluated and presented geographical information data of proposed sites for 2030. Whereas the majority of the year 2020 healthcare facilities are primary, majority of the proposed sites for 2030 are secondary and tertiary. This indicated that Ghana requires more quality healthcare facilities. Ghana as precedent for the SSA region, it was evaluated to leave no disease behind to reinforcement the SDG 3 health target of leaving no one behind that target’s UHC. In the current surveillance state, community-based surveillance system was proposed over healthcare facility-based surveillance. This is to collect health information on the broad spectrum of IDs as the principle to leave no disease behind in Ghana and SSA. For future benefits, the study recommends primary healthcare facilities’ expansion in under-served areas to cater for the surveillance of ERIDs across the country.

## Abbreviations

CHAG: Christian Health Association of Ghana
CHPS: Community-based Health Planning and Services
COVID-19: Coronavirus Disease 2019
ERID: Emerging and Re-emerging Infectious Disease
GHS: Ghana Health Service
GHS-SMOD: Global Human Settlement Model Grid
GIDEON: Global Infectious Diseases and Epidemiology Network
ID: Infectious Disease
PPMED: Policy Planning Monitoring and Evaluation Division
ProMED: Program for Monitoring Emerging Diseases
QGIS: Quantum Geographic Information System
SDG: Sustainable Development Goals
SSA: sub-Saharan Africa
UHC: Universal Health Coverage
WGS84: World Geodetic System 1984
WHO: World Health Organisation
